# Searching for a Measure Integrating Sustainable and Healthy Eating Behaviors

**DOI:** 10.3390/nu11010095

**Published:** 2019-01-05

**Authors:** Sylwia Żakowska-Biemans, Zuzanna Pieniak, Eliza Kostyra, Krystyna Gutkowska

**Affiliations:** 1 Department of Organization and Consumption Economics, Faculty of Human Nutrition and Consumer Sciences, Warsaw University of Life Sciences WULS-SGGW, Nowoursynowska 159C, 02-776 Warsaw, Poland; krystyna_gutkowska@sggw.pl; 2 Consumer and Sensory Research Institute Ltd., Sienna 55/9, 00-820 Warsaw, Poland; zlpieniak@gmail.com; 3 Department of Functional Food, Ecological Food and Commodities, Faculty of Human Nutrition and Consumer Sciences, Warsaw University of Life Sciences WULS-SGGW, Nowoursynowska 159C, 02-776 Warsaw, Poland; eliza_kostyra@sggw.pl

**Keywords:** food, young adults, scale development, sustainability, health

## Abstract

Sustainable and healthy food-related behavior is high on the public policy and research agenda due to its potential to cope with negative environmental and health outcomes. There are several measures related to sustainability in food choices but there have not been many attempts to integrate sustainable and healthy eating (SHE) behaviors into one measurement instrument so far. Therefore, the main aim of this study was to identify how young adults interpret the SHE concept and to develop an instrument that measures a self-reported consumer’s SHE behavior. The process of scale development involved an exploratory qualitative study and two quantitative studies. As a result of 20 individual in depth interviews with Polish young adults, 50 items were generated reflecting their perspective on principles of SHE (Study 1). Two samples were used in the scale validation process: *n* = 217 (Study 2) and *n* = 220 (Study 3). Via principal component analysis, reliability analysis, and confirmatory factor analysis, the final form of the scale was derived. The proposed 34-item scale offers insights into the most relevant aspects of SHE behaviors, grouped in eight factors: “healthy and balanced diet”, “certification and quality labels”, “meat reduction”, “selection of local food”, “choice of low fat food products”, “avoidance of food waste” and purchase and consumption of food products that are respecting “animal welfare” and finally choice of “seasonal food”. Although the developed scale can benefit from further refinement and validity testing in different cultural and social background, it is clear that the scale, as developed, can be a useful tool for researchers who are interested in the study of SHE behaviors.

## 1. Introduction

Food consumption is one of the most important drivers of environmental pressures, and the adoption of healthy diets is suggested to be an option for less environmentally intensive food habits and improved public health [1,2]. Linking food and eating to sustainability brings a new dimension that well reflects consumers’ changing values and lifestyles. With increasing recognition of the environmental impact of food and drink, food policy, dietary recommendations, and research on consumer behavior need to go beyond the traditional focus on healthy eating and include wider issues of social, environmental, and economic sustainability [3,4]. At present, dietary guidelines worldwide are still focused principally on health, but an increasing number of nutrition and public health experts are suggesting that future dietary guidelines should combine both environmental and nutritional aspects [5,6]. Therefore, more attention has been paid to incorporating the idea of sustainability with the promotion of healthy eating, but very few policy recommendations exist [7,8]. In some countries, the sustainability aspect has already become an integral consideration in the current dietary recommendations. In 2014, the FAO/WHO 2nd International Conference on Nutrition devoted 9 of its 60 recommendations to actions for sustainable food systems promoting healthy diets. Some EU countries (e.g., Sweden, The Netherlands, and the United Kingdom) made attempts to incorporate sustainable and healthy eating (SHE) principles into dietary guidelines. However, the proposals were not adopted more broadly due to the EU members’ fears of erecting trade barriers related to favoring locally produced food or products complying with additional standards like fair trade [3,9].

Consumer behavior toward healthy eating and adherence to the dietary recommendations have been widely explored [10,11,12]. However, a more holistic view of sustainable healthy eating behavior has received less attention, albeit more consumer research is emerging in this area [4,13,14]. Consumer research has previously focused mainly on specific areas of sustainable food, such as organic food [15,16,17], local or traditional food [18,19], ethical food purchases [20], meat substitution, and meat reduction [1,21,22]. Despite the paramount importance of consumer demand in shaping the realization of sustainable diet recommendations, far fewer studies have assessed consumer preferences for sustainable dietary alternatives compared with studies that have assessed diets’ environmental impacts [4].

In order to achieve more sustainable diets, research is therefore required on both what a sustainable diet is and how more sustainable eating habits can be introduced in society [23]. Barilla Center for Food and Nutrition (BCFN) recently developed the model of the Double Pyramid on Food and Environment to illustrate the relationship between a healthy diet and environmental impact [24]. The model visualize two pyramids. The first is the Food Pyramid based on the Mediterranean Diet, and the second one is inverted and reclassifies foods according to their environmental impact. 

There is no doubt that a healthy diet is largely a sustainable diet [5,25,26]. However, some possible trade-offs and synergies of combing the aspects of healthy eating with sustainability from a consumer point of view exist as well [27]. The social and economic implications are as important as health and environment if moves toward sustainable consumption patterns are to materialize [28].

### 1.1. Sustainable Eating Concepts

There have been several attempts to determine the principles of a sustainable healthy diet, but there is no consensus on what a sustainable and healthy diet is comprised of [2,29,30,31]. Sustainable consumption usually refers to a level and pattern of consumption that meets the needs of the present without compromising the ability of future generations to meet their needs [32]. Environmental sustainability is defined by Goodland [33] as “the maintenance of nature capital”. The Brundtland Commission Report [32] presents the most widely accepted definition of sustainability: “meeting the needs of the present without compromising the ability of future generations to meet their needs”. Similarly, the Norwegian Ministry for the Environment (1994) defined sustainable consumption “as the use of services and related products which respond to basic needs and bring a better quality of life while minimizing the use of natural resources and toxic materials as well as the emissions of waste and pollutants over the life-cycle so as not to jeopardize the needs of future generations” [34]. In some studies, a reference is made directly to the investigated topic. For instance, Austgulen [29] states that the “sustainable consumption of meat -must address- concerns related to the environment with a special focus on climate change since livestock production is a significant contributor to climate change”. In public health nutrition, sustainability refers to the ability to maintain food system capacity to support the nutritional health needs of current and future populations, while protecting the ecological systems that produce food [35]. The Food and Agriculture Organization of the United Nations [36] made one of the first attempts to tie up sustainability with a human diet and health, defining sustainable diets as “diets with low environmental impacts which contribute to food and nutrition security and to a healthy life for present and future generations. Sustainable diets are protective and respectful of biodiversity and ecosystems, culturally acceptable, accessible, economically fair and affordable; they are nutritionally adequate, safe and healthy, while optimizing natural and human resources”. 

Another approach to linking sustainability with healthy eating was presented within the “LiveWell for Low Impact Food in Europe” (LIFE) project implemented by The World Wide Fund for Nature (WWF) [37]. A sustainable diet is regarded as “a healthy, low-carbon diet that takes account of cultural preferences. Its focus is on mitigating greenhouse gas emissions, but it incorporates health, socio-cultural, economic and qualitative elements as well”. The LiveWell project promoted six basic principles guiding sustainable and healthy food related to behaviors such as (1) increased consumption of fruit and vegetables, (2) eating a variety of foods, (3) avoiding wasting food, (4) moderating meat consumption, (5) buying certified food, and (6) eating fewer foods high in fat, salt, and sugar and avoiding sugary drinks [37].

In this study, we applied both the FAO definition of “sustainable diet” and the LiveWell approach and principles of SHE behaviors that were explored further within the exploratory phase of scale development (i.e., qualitative, individual in-depth interviews with young adults). 

### 1.2. Measures of Sustainable Food-Related Behaviors

There exist several validated measurement scales for assessing various aspects of sustainable food-related behaviors (e.g., pro-ecological behavior, index of sustainability of food practices, sustainable food behavior, or green eating behavior scale) (Table 1).

Index of sustainability of food practices, developed by Tobler and colleagues [38], investigated different ecological food consumption patterns that consumers are willing to adopt. Verain et al. [31] empirically explored different types of sustainable food behaviors. A distinction between sustainable product choices and curtailment behavior has been made and predictors of the two types of behavior have been identified. Weller et al. [39] developed and validated an instrument to access environmentally conscious eating, so called Green Eating Behavior. Monoroe et al. [40] examined the original Green Eating behavior scale consisting of six items assessing the frequency of pro-environmental food choices. 

Therefore, the main aim of the study was to develop an instrument that measures a self-reported consumer’s SHE behavior. This study belongs to a broader research project aimed at assessing SHE behaviors of young adults funded by the National Science Centre in Poland. 

## 2. Materials and Methods

### 2.1. The Scale Development Process

Data collection process involved (1) a qualitative study using IDI to identify how consumers conceptualize the term “sustainability”, and in relation to healthy eating, and (2) two quantitative studies to validate the measures developed in the exploratory phase of the research (Figure 1). All subjects gave their informed consent for inclusion before they participated in the study. The study was conducted in accordance with the Declaration of Helsinki, and the protocol was approved by the Ethics Committee of the Food and Nutrition Institute in Warsaw, Poland, on 29 December 2015 (Project No. 2013/10/M/HS4/00477).

The target group of this study was young adults (aged 18–30), since young adults often establish unfavorable dietary habits when leaving the parental home, which may have an impact on their health [41]. 

### 2.2. Study 1: Item Generation and Screening—Exploratory Study

Twenty individual in-depth interviews (IDIs) with young adults were carried out in order to obtain insights on consumers’ attitudes, knowledge, and perception, as well as current behavior toward sustainable healthy eating. The snowball sampling technique has been used to recruit the participants. First, respondents were asked to give free association of the term “sustainability” and its link with food and healthy eating. Then, based on the extensive literature review performed prior to the qualitative study [30,31,34] and our current understanding, six principles of SHE introduced in the campaign “LiveWell” [37] were used as a starting point to discuss different elements of sustainable healthy eating with the interviewees. Interviews ranged from approximately 60 to 90 min in length and were audiotape-recorded. The data analysis involved coding the interview material and constructing conceptual categories from the emerging codes. 

### 2.3. Study 2: Pre-Test (Scale Purification)

Based on the literature review and information collected through IDIs, a preliminary self-administered questionnaire was developed and tested on a judgment sample (a non-probability sample comprised of individuals with judgments and attitudes about the subject matter being researched) of 217 young adults in May 2016 using PAPI (pen and paper interviews) method. This questionnaire consisted of 50 items and covered a wide range of various behaviors that have been recognized in previous research and emerged from IDIs, including adherence to dietary recommendations (e.g., five portions of fruit and vegetables a day, low salt and sugar consumption, a varied and balanced diet, consumption of processed food), the purchase of organic, local, and seasonal foods, meat- and plant-based food consumption, and sustainability aspects (e.g., food waste). For details, see Appendix A. A seven-point frequency scale ranging from (1) never to (4) sometimes to (7) always was used. Items were purified based on the examination of the results of the exploratory factor analysis (EFA), through varimax rotation, the average corrected item-to-total correlation, and internal reliability analysis through Cronbach’s alpha.

### 2.4. Study 3: Scale Validation

Study 3 was designed to assess the replicability of the eight factors in a new test over a four-week period. Quantitative descriptive data for Study 3 were collected through a cross-sectional consumer survey in June 2016. Total sample size was 220 respondents. A stratified design was used to recruit 18–30-year-old adults. The sample was representative in terms of gender and region of the Polish population. Participant recruitment of the sample and survey was done by a professional market agency in accordance with ICC/ESOMAR—International Code of Conduct for Market and Social Research. Highly qualified and experienced interviewers carried out face-to-face interviews at home using a CAPI approach (computer-assisted personal interviewing). A seven-point frequency scale ranging from (1) never through (4) sometimes to (7) always was used. Demographic characteristics, including marital status, occupation, education, income, and eating habits (vegetarian etc.), were also collected. The profiles of the respondents that participated in Study 2 and Study 3 are included in Table 2.

### 2.5. Statistical Analysis

Data were analyzed using the statistical software SPSS version 23.0PL (SPSS Inc., Chicago, IL, USA) and LISREL 9.2 (SSI Scientific Software Int.). Missing data were imputed by means of the EM (expectation-maximization) algorithm. First, an exploratory factor analysis using the principal components extraction method with varimax rotation was performed on data from Study 2. The purpose of the exploratory factor analysis is to identify a set of latent (thus unobserved) constructs (here referred to as factors) underlying a battery of observed variables (the 50 proposed sustainable healthy eating items). It is commonly used by researchers when developing a scale [42]. 

Next, maximum likelihood confirmatory factor analysis was performed using the robust maximum likelihood procedure in LISREL 9.2 (Study 2 and Study 3). The reason to perform it was to determine whether measures of a construct actually converge the intended latent variable or share a high proportion of variance in common (convergent validity) and whether the constructs are distinct from each other (discriminant validity). To evaluate the model fit, the ratio of chi-squared to degrees of freedom (χ^2^/df) are reported, as well as three other indices: the root mean square error of approximation (RMSEA), the normed fit index (NFI), and the comparative fit index (CFI). Values below 0.08 for RMSEA [43] and above 0.90 for NFI and CFI [44] and values under 5 for χ^2^/df [36] indicate an acceptable fit. 

## 3. Results

### 3.1. Perception of the Term “Sustainability” and “Sustainable Healthy Eating” by Young Adults (Study 1)

The results of the IDIs showed that young Polish consumers associated the term “sustainability” with maintaining balance between private life and work, between physical and mental (spiritual) well-being, effective management of their budget, and activities related to environmental protection. 

Respondents were further asked about their associations with the term “sustainable food” (see Table 3). In general, sustainable food has a very positive image among Polish young adults and it has been directly associated with healthiness, balance, and varied eating behavior. It was also perceived as more expensive and less available in comparison with non-sustainable food.

Further exploration of the sustainability in relation to food led to defining the term “sustainable healthy eating” that has been associated with a balanced and varied diet, adherence to dietary recommendations, and, in general, taking care of one’s health, including physical activity. Participants perceived sustainable healthy eating as providing necessary vitamins and minerals. Additionally, food production methods and the origin of food, such as organic food, an environmentally friendly production methods, and local production, have been associated with sustainable healthy eating. Respondents conceptualized SHE referring to food that has a country of origin clearly marked on its label. Finally, sustainable healthy eating was associated with the consumption of food that is natural, fresh, without additives, and having undergone a low amount of processing. These perceptions and association about sustainable healthy eating served as a basis to create items to be assessed in the quantitative phase of research (Study 2 and Study 3).

### 3.2. Results of Exploratory Factor Analysis in Study 2

The 50 items of the proposed measure of sustainable healthy eating developed within Study 1 were factor analyzed with varimax rotation. Various solutions were considered and an eight-factor solution fit the data best. Items with a severely skewed distribution and those which did not load clearly on a single factor were rejected. After following this procedure, 34 items were left. 

Final explanatory factor analysis performed on those items explained in total 64.7% of the variation in the data and with eigenvalues ranging from 9.66 to 1.04. The selection of factors was based on eigenvalues (>1 as threshold), while factor loadings were used to interpret the meaning of the resulting factors and term them accordingly (Table 4). *Factor 1* consists of 10 statements related to the choice of food that is healthy, nutritious, natural, and in general indicating a balanced diet and is therefore labeled as a “healthy and balanced diet”, while *Factor 2* contains five items related to aspects such as regional certificate, organic food, and quality marks on labels. The fifth item loaded on three different factors, but the highest factor loading was for this factor. Additionally, previous studies show that organic food is commonly associated with protection of the environment that is also reflected in the hierarchy of motives to buy organic food [4,37,38], so this item was finally allocated to this factor. *Factor 2* is consequently termed as “quality labels (regional and organic)” reflecting consumer interest in food products with distinctive features such as regional and organic food. *Factor 3* is composed of four statements related to the replacement of meat by a plant–protein source food products and in general meat reduction and was therefore called “meat reduction”. *Factor 4* consists of three items, refers to the concept of locally produced foods, and is labeled as “local foods”. *Factor 5* includes three items related to the consumption of food products that are low in fat and is termed “low fat”. *Factor 6* has three items related to not wasting food and is named “avoiding food waste”. *Factor 7* consists of three factors related to the choice of free range eggs and sustainable fishing and is therefore considered to be an “animal welfare factor”. The factor loading of the last item scored below the advised value of 0.60, but we believe that it contributes to the overall meaning of this factor. Additionally, the Cronbach’s alpha was acceptable for this factor. *Factor 8* includes three statements about the consumption of seasonal food products and is labeled as “seasonal food”. Here again, the factor loading of the last item was relatively low; however, taking into account the general meaning of this item and the Cronbach’s alpha score, it was included in the final factor. No factors with a logical aspect composition were found. Cronbach’s alpha internal reliability coefficients ranged from 0.60 to 0.92, above the threshold value for satisfactory scales.

The intercorrelations between the factors are presented in Table 5. Almost all correlation coefficients were significant (*p* < 0.01) but below 0.7, so (severe) multicollinearity is not a concern in the present data [45]. The most prominent correlation was between *healthy & balanced diet* and *quality labels* (*r* = 0.567). Most of the correlations were moderate. Only the correlations between Factor 6 (*avoiding food waste*) and Factor 3 (*meat reduction*) and between Factor 6 and Factor 5 (*low fat*) were not significant.

### 3.3. Confirmatory Factor Analysis of the Items Measured in Study 2 and Study 3

Standardized factor loadings for both Study 2 and Study 3 are presented in Table 6. The χ^2^ for the model for Study 2 (*n* = 217) was 870.57 with 499 degrees of freedom (*p* < 0.001), and the ratio χ^2^/df of 1.74. The RMSEA value was 0.059, the NFI was 0.91, and the CFI was 0.96, so all are acceptable. Moreover, the other goodness-of-fit indices were satisfactory. Hence, it may be concluded that the model presented in Table 6 fits the data well. 

Almost all individual item standardized factor loadings on the factors were highly significant with values ranging from 0.52 to 0.93. Two-item loadings were relatively low, particularly 0.49 (“I limit my salt usage”) and 0.49 (“I try not to throw away food”). Nevertheless, we have decided to keep the items in the respective factors because of their important theoretical input to the factors and whole scale. Further analysis has shown that including those items still revealed “good” constructs. No cross loadings of 0.4 or more appeared. Hence, all other items were also considered in the interpretation of the factors [46]. This analysis confirms the results of the exploratory factor analysis presented in Table 4. 

The eight-factor model of Study 2 provided a good fit for the data collected in Study 3. The χ^2^ for this model was 1105.52 with 499 degrees of freedom (*p* < 0.001), and the ratio χ^2^/df of 2.21. The RMSEA value was still acceptable 0.074, the NFI was 0.96, and the CFI was 0.98, so both are very good. Moreover, the other goodness-of-fit indices were satisfactory. All parameters estimates were significant at *p* < 0.001. The standardized factor loadings were in general higher than in Study 2 and ranged from 0.62 to 0.93. The two items that previously loaded low had satisfactory scores in this analysis. However, two other items loaded relatively low—0.46 (*Whenever possible, I choose fruits and vegetables from my own allotments (plots)*) and 0.33 (*I eat seasonal fruits and vegetables*)—but were kept in the scale due to their meaning and overall fitting within the factors. These analyses indicate that the factor structure of the sustainable healthy eating scale identified in Table 4 was confirmed in the independent Study 3 sample.

### 3.4. Comparison of the Mean Values between Study 2 and Study 3

Mean values for different factors of the sustainable healthy eating resulting from confirmatory factor analysis are presented in Table 7. In general, respondents from both Study 2 and Study 3 most frequently avoided food waste, followed by the consumption of seasonal food. The consumption of local food, low fat food, and a healthy and balanced diet scored close to the neutral point of the scale. In both studies, respondents were least frequently reducing meat consumption. 

## 4. Discussion

This study is an attempt to measure SHE behaviors, taking into account results from an exploratory qualitative study, current definitions of sustainability, and existing scales related to sustainable (and healthy) eating behavior.

Sustainable healthy eating is a complex, multidimensional concept, so the developed measure is of a multidimensional nature and includes items related to pro-ecological behavior, such as the avoidance of food waste, the consumption of local and seasonal foods, or taking into consideration aspects of animal welfare, as well as aspects related to pro-healthy eating, such as consumption of healthy and balanced diet, food products that are low in fat or reduction of meat consumption. One additional aspect has been shown to be important in an assessment of SHE: the usage of “quality labels”, including regional and organic certificates. Healthy eating patterns, an interest in cooking, and supporting environmental policy measures were all positively correlated to sustainable food consumption, which confirms findings of Barone et al. [47].

A *healthy and balanced diet* is the biggest factor and a very broad concept including 10 items related to choosing food that is natural, healthy, and nutritious as well as food rich in vitamins and minerals, information that is thus largely communicated not only by public health authorities but also by the food industry in advertisements and every day messages. Interestingly, behavior related to limiting salt and sugar consumption loaded on the same factor as statements related to a generally healthy and balanced diet, whereas a choice of low fat products loaded on a separate factor: *low fat*. This might be a result of previous dietary recommendations that were communicated in Poland. The focus was on lowering fat consumption without making a clear distinction between animal- and plant-based fat. It is important to remember that still, three decades ago, Polish people were consuming mostly animal fat. The changes in food consumption followed changes in economic policy (in 1990), including reductions in subsidies for dairy and other animal fats, and a free market provided relatively cheap vegetable oils. Additionally, public health initiatives promoted the consumption of plant-based fat (mainly rapeseed oil) instead of animal fat. This was a successful initiative resulting in changes in dietary habits among most consumers followed by a fall in mortality due to coronary heart disease by 24% over five years [48,49]. Afterwards, a new trend introducing all the “light products” was present not only in Poland but also in other European countries. All of this might explain why consumption of low fat products loaded as a separate factor in our data. *Quality labels (regional and organic)* is the factor with the second-highest amount of items. Knowledge of sustainability labels (such as regional certificates, organic labels, and quality labels) makes it feasible for consumers to apply SHE principles in their daily life [50]. Consumers with preferences for environmental attributes can only adjust consumption patterns in line with these preferences if environmentally sustainable products can be identified at the point of purchase [51]. Therefore, this contributes to a better understanding of consumers’ sustainability related food choices and behaviors. *Meat reduction* is the third factor and consists of three statements related to the replacement of meat by pulses and only one item stating the avoidance of meat. Van Loo et al. [14] found that a diet with a higher proportion of products of plant origin is associated with a healthy and sustainable diet. Vegetarian and vegan diets that exclude meat and/or all animal products have been shown to have lower dietary emissions [2,52]. Plant-based meat substitutes have also been identified as healthy sources of protein that, in comparison to meat, offer a number of social, environmental, and health benefits and may play a role in reducing meat consumption [53]. Reduced meat consumption is expected to have a positive effect on public health due to the reduced intake of saturated fat, but from a nutrition perspective restriction in meat consumption is most critical for the intake of iron and zinc [1]. Therefore, to promote a dietary change toward SHE behaviors, both aspects should be considered and further analyzed to develop recommendation on meat reduction. 

It is interesting to notice that items included in factor eight, namely *seasonal food*, relate to the recommendation to eat five portions of fruit and vegetables per day, which is based on advice from the World Health Organization to eat a minimum of 400 g of fruit and vegetables a day to lower the risk of serious health problems, such as heart disease, stroke, and some types of cancer [54]. In Poland, a high consumption of fruits and vegetables is associated with local and seasonal products sold at farmer’s markets. It is likely that those who consume a high amount of fruits and vegetables buy them at farmer’s markets. Therefore, this association of fruit and vegetables with seasonal food products well reflect consumers’ perceptions [28]. The seventh factor relating to the purchase and consumption of food products that are respecting *animal welfare* consists only of items linked to the purchase of free-range eggs and fish from sustainable fisheries. These statements are in line with the Green Eating behavior scale [39], where items related to the frequency of purchasing meat or poultry labeled “free range”. *Food waste* emerged as a particular policy hotspot in contemporary societies, and reducing waste has been a persistent goal of food policy since the 1930s [55]. Activities related to avoiding food waste and consumption of seasonal and local food were reported as the most frequently done by Polish young adults. The local food system is considered a great entry way to connect sustainability and health, leveraging co-benefits for both planet and people [56]. These findings are similar to those by Tobler et al. [38], who found that buying local food and avoiding excessive packaging were the most popular activities, whereas reducing meat consumption was the least popular. 

This multidimensional scale provides a broad spectrum of behaviors that can be included under the concept of sustainable healthy eating. Existing scales presented in Table 1 referred mostly to sustainable behavior without taking into account the aspects related to the principles of healthy eating. Our scale addressed both aspects and includes consumers’ perspective on SHE principles. Therefore, our results can contribute to a better understanding of how consumers interpret and translate these concepts into food choices. It also adds value to the existing knowledge since it proves that eating sustainably and eating healthily are complementary concepts in a consumer’s mind. However, these results are preliminary, and there are several study limitations that should be addressed in further research. First, this study focused only on a target group of young Polish adults, which limits the potential of generalizing our findings. Future studies should focus on further validation of the proposed measure among populations with different cultural, political, and religious backgrounds. Second, the present study, like most of the research in this field, builds on self-reported behavior. Although such self-reported and subjective opinions provide valuable insights into factors shaping consumer behavior, several of the self-reported variables can be regarded as specifically prone to social desirability bias and hence may deviate from actual behavior [57]. Finally, the formulation of the items covered in the proposed measure was based on the in-depth interviews carried out with the target group and were directing respondents’ attention to sustainable as well as healthy eating considerations when responding about their behavior. They may behave in a certain way with respect to food for many different reasons [38]. 

## 5. Conclusions

Promoting food sustainability requires much more attention to cultural and social contexts and the food philosophies of specific groups of consumers [58]. It is important that consumers’ involvement in SHE be translated into actual behavior with the help of well-designed communication strategies. The result of our study with young adults revealed that SHE is a multidimensional construct with eight core dimensions representing consumers concerns of eating healthily while respecting principles of sustainability in terms of reducing environmental impact of food production and distribution. This study confirms that the concept of a healthy diet and that of a sustainable diet are complementary in a consumer’s mind. From a public health policy perspective, identifying SHE behaviors may help to encourage consumers to engage in responsible food consumption in terms of both health and environmental impacts. Overall, the results of the present study provide researchers and professionals interested in this field with a measure for assessing SHE that should be further validated in other studies with greater sample sizes and in different cultural, political, and religious backgrounds.

## Figures and Tables

**Figure 1:** The scale development process.

**Table:** 

**Study 1** **Exploratory phase** IDIs (individual in-depth interviews) *n* = 20 identification of 50 items	**Study 2** **Pre-test (scale purification)** PAPI (pen and paper interviews) survey *n* = 217 exploratory factor analysis confirmatory factor analysis 34-item scale	**Study 3** **Scale validation** CAPI (computer assisted personal interviews) survey *n* = 220 confirmatory factor analysis

**Table 1:** Overview of scales used to measure sustainable eating behavior.

Name of the Scale	Source	Items	Scale
Index of sustainability of food practices	Tobler, C., Visschers, V.H.M., & Siegrist, M. (2011). Eating green. Consumers’ willingness to adopt ecological food consumption behaviors. Appetite, 57, 674–682	Buy regional (local) food Avoid products with excessive packaging Buy organic food Eat only seasonal fruit and vegetables Eat meat at most twice a week or a little at a time Avoid food products that were imported by airplane	4-point scale: (1). I am doing this already; (2). I would like to do this and already know how to start; (3). I would like to do this, but I do not know how; (4). I am not doing this and I am not willing
Sustainable food behavior	Verain, M., Dagevos, H., Antonides, G. (2015). Sustainable food consumption. Product choice or curtailment? Appetite, 91, 375–384	Buying organic meat Buying organic fruits and vegetables Buying organic dairy Buying free range meat Buying products with a sustainability label Eating smaller portions of meat Eating less Eating less dairy One meat free day a week	Dichotomous scale “yes” and “no”
Green Eating behavior scale	Weller, K.E.; Greene, G.W.; Redding, C.A.; Paiva, A.L.; Lofgren, I.; Nash, J.T.; Kobayashi, H. (2014). Development and validation of Green Eating behaviors, stage of change, decisional balance, and self-efficacy scales in college students. Journal of Nutrition Education and Behavior, 46 (5), 324–333.	Locally grown foods are grown within 100 miles from your location. Based on this, how often do you eat locally grown foods? When in season, how often do you shop at farmer’s markets? How often do you choose foods that are labeled USDA organic? How often do you select meats, poultry, and dairy products that are raised without antibiotics or hormones? How often do you select food or beverages that are labeled fair-trade certified? How often do you buy meat or poultry labeled “free range” or “cage free”?	5-point scale: (1) barely ever to never, rarely (25%), sometimes (50%), often (75%), almost always

**Table 2:** Overview of the samples in Study 2 and Study 3.

	Study 2		Study 3	
*Gender*	N	%	N	%
Female	116	53.4	104	47.3
Male	101	46.6	116	52.7
*Age*				
18–24	217	100	105	4.7
25–30	-	-	115	52.3
*Education*				
Primary	-	-	24	10.9
Vocational, lower secondary school	-		22	10.0
Secondary	-	-	113	51.4
College, University and above	217	100	61	27.7
*Place of residence*				
Up to 99 thousand inhabitants	-	-	59	26.8
100–499 thousand inhabitants	-	-	98	44.5
Above 500 thousand inhabitants	-	-	63	28.6

**Table 3:** Associations with the term “sustainable food” in Study 1.

Sustainable Food	Non-Sustainable Food
Healthy, balanced and varied eating behavior, higher consumption of fruits and vegetables, not too fatty Fresh, natural Tasty Regular meal consumption Organic production, short expiry date, known origin Good for mood: well-being, happiness Expensive (higher price) Environmental friendly, safe Local deliveries Lower availability	Not healthy, conventional food products, less natural (artificial), fertilized, “clinical” (clinically produced; plastic-looking), with additives, highly processed Artificial ingredients, preservatives, sweets, fast food No regularity in consumption (e.g., eating at night), inadequate diet (e.g., too much fat), less varied (fatty and sweet food) Lower price High availability

**Table 4:** Items, factor loadings, and Cronbach’s alpha of the exploratory factor analysis in Study 2.

Factors	Items	Factor Loadings	Cronbach’s Alpha
Factor 1	HEALTHY & BALANCED DIET		0.880
	I choose food that is nutritious.	0.773	
	I choose food that keeps me healthy.	0.714	
	I avoid sugary drinks.	0.672	
	I choose food that contains a lot of vitamins & minerals.	0.644	
	I choose food that contains natural ingredients.	0.616	
	I choose food that contains no additives.	0.599	
	I try to have a balanced diet.	0.585	
	I choose food that contains no artificial ingredients.	0.575	
	I choose whole grains products.	0.550	
	I limit my salt usage.	0.547	
Factor 2	QUALITY LABELS (REGIONAL AND ORGANIC)		0.800
	I choose food products with a regional certificate.	0.807	
	When buying food, I check certificates and quality marks on labels.	0.733	
	Whenever possible, I buy organic food.	0.624	
	I buy regional food.	0.585	
	I choose food that is produced in an environmental friendly way.	0.498	
Factor 3	MEAT REDUCTION		0.848
	Pulses replace meat in my cooking.	0.858	
	I try to eat as many pulses as possible in order to reduce meat consumption.	0.790	
	I try to eat as much plant–protein source food products as possible, e.g., pulses.	0.745	
	I avoid eating meat.	0.734	
Factor 4	LOCAL FOOD		0.772
	I buy fruits and vegetables directly from the farmer.	0.757	
	Whenever possible, I choose fruits and vegetables from my own allotments (plots).	0.729	
	I buy locally produced foods.	0.679	
Factor 5	LOW FAT		0.923
	Whenever possible, I choose low fat food products.	0.901	
	I choose low fat food products.	0.873	
	I avoid food products containing lots of fat.	0.863	
Factor 6	AVOIDING FOOD WASTE		0.601
	I don’t waste food.	0.742	
	I try not to throw away food.	0.708	
	I reuse leftovers from food.	0.656	
Factor 7	ANIMAL WELFARE		0.681
	I choose free-range eggs.	0.769	
	I avoid buying battery eggs.	0.775	
	Whenever possible, I buy fish from sustainable fishing.	0.479	
Factor 8	SEASONAL FOOD		0.674
	I eat five portions of fruits and vegetables a day.	0.754	
	In season, I shop at farmer’s market.	0.584	
	I eat seasonal fruits and vegetables.	0.469	

**Table 5:** Intercorrelations between different factors of the sustainable healthy eating concept.

	1	2	3	4	5	6	7	8
1. Healthy balanced diet	1							
2. Quality labels	0.567 *	1						
3. Meat reduction	0.405 *	0.392 *	1					
4. Local foods	0.370 *	0.478 *	0.314 *	1				
5. Low fat	0.428 *	0.280 *	0.300 *	0.186 *	1			
6. Avoiding food waste	0.319 *	0.254 *	0.098	0.223 *	0.094	1		
7. Animal welfare	0.359 *	0.381 *	0.339 *	0.474 *	0.190 *	0.184 *	1	
8. Seasonal food	0.475 *	0.341 *	0.330 *	0.440 *	0.258 *	0.244 *	0.324 *	1

* *p* < 0.01.

**Table 6:** Standardized factor loadings for Study 2 and Study 3 (Confirmatory Factor Analysis).

Factors	Items	Study 2	Study 3
		Standardized Factor Loadings	
Factor 1	HEALTHY & BALANCED DIET		
	I choose food that is nutritious.	0.775	0.785
	I choose food that keeps me healthy.	0.778	0.839
	I avoid sugary drinks.	0.622	0.698
	I choose food that contains a lot of vitamins & minerals.	0.739	0.819
	I choose food that contains natural ingredients.	0.683	0.753
	I choose food that contains no additives.	0.643	0.767
	I try to have a balanced diet.	0.614	0.781
	I choose food that contains no artificial ingredients.	0.651	0.823
	I choose whole grains products.	0.587	0.768
	I limit my salt usage.	0.491	0.690
Factor 2	QUALITY LABELS (REGIONAL AND ORGANIC)		
	I choose food products with regional certificate.	0.643	0.754
	When buying food, I check certificates and quality marks on labels.	0.592	0.745
	Whenever possible, I buy organic food.	0.695	0.773
	I buy regional food.	0.608	0.696
	I choose food that is produced in an environmental friendly way.	0.753	0.879
Factor 3	MEAT REDUCTION		
	Pulses replace meat in my cooking.	0.826	0.877
	I try to eat as many pulses as possible in order to reduce meat consumption.	0.830	0.892
	I try to eat as much plant–protein source food products as possible, e.g., pulses.	0.754	0.845
	I avoid eating meat.	0.654	0.780
Factor 4	LOCAL FOOD		
	I buy fruits and vegetables directly from the farmer.	0.754	0.682
	Whenever possible, I choose fruits and vegetables from my own allotments (plots).	0.715	0.457
	I buy locally produced foods.	0.726	0.875
Factor 5	LOW FAT		
	Whenever possible, I choose low fat food products.	0.917	0.927
	I choose low fat food products.	0.925	0.897
	I avoid food products containing lots of fat.	0.840	0.885
Factor 6	AVOIDING FOOD WASTE		
	I don’t waste food.	0.558	0.670
	I try not to throw away food.	0.492	0.732
	I reuse leftovers from food.	0.667	0.691
Factor 7	ANIMAL WELFARE		
	I choose free-range eggs.	0.708	0.869
	I avoid buying battery eggs.	0.613	0.821
	Whenever possible, I buy fish from sustainable fishing.	0.627	0.648
Factor 8	SEASONAL FOOD		
	I eat five portions of fruits and vegetables per day.	0.515	0.657
	In season, I shop at farmer’s market.	0.797	0.622
	I eat seasonal fruits and vegetables.	0.639	0.325

Fit-statistics for Study 2: χ^2^ (499) = 870.57, *p* < 0.001; RMSEA = 0.059; NFI = 0.91; CFI = 0.96 (*n* = 217). Fit-statistics for Study 3: χ^2^ (499) = 1105.52, *p* < 0.001; RMSEA = 0.074; NFI = 0.96; CFI = 0.98 (*n* = 220).

**Table 7:** Mean values and standard deviations of the factors resulting from Study 2 and Study 3 (Confirmatory Factor Analysis).

Factors	Study 2		Study 3	
	Mean	St. Dev.	Mean	St. Dev.
1. Healthy & balanced diet	4.17	1.06	4.03	0.96
2. Quality labels	3.18	1.17	3.89	1.18
3. Meat reduction	2.44	1.28	3.36	1.45
4. Local food	4.32	1.59	3.98	1.20
5. Low fat	4.07	1.62	4.42	1.40
6. Avoiding food waste	4.92	1.16	4.67	1.16
7. Animal welfare	4.39	1.56	3.91	1.52
8. Seasonal food	4.50	1.20	4.59	0.97

## Appendix A. 50 Statements Used in the Scale Development Process

Please read the statements carefully and indicate your answer on a seven-point scale where 1 is never, 4 is sometimes, and 7 is always.

	**Statement**
1	I eat five portions of fruits and vegetables a day.
2	I choose whole grains products.
3	I choose products from varied food groups.
4	I try to have a colorful plate.
5	I choose food that contains no additives.
6	I choose food that contains natural ingredients.
7	I choose food that contains no artificial ingredients.
8	Whenever possible, I buy organic food.
9	I read information placed on the product label.
10	When buying food, I check certificates and quality marks on labels.
11	I choose products with clear information/indication on country of origin.
12	I choose food products with regional certificate.
13	I pay attention to the expiry date while shopping.
14	I don’t waste food.
15	I reuse leftovers from food.
16	I don’t eat meat once a week.
17	I try not to eat meat at least once or twice a week.
18	I buy regional food.
19	I eat seasonal fruits and vegetables.
20	In season, I shop at farmer’s market.
21	I choose low processed food.
22	I drink enough water (2 L) throughout a day.
23	I avoid sugary drinks.
24	I limit my salt usage.
25	I keep food such as cakes, sweets and chocolate to an occasional treat.
26	I keep food such as fries and crisps to an occasional treat.
27	I limit my consumption of dry-cured meats/cold meats.
28	I choose food that contains a lot of vitamins & minerals.
29	I choose food that keeps me healthy.
30	I choose food that is nutritious.
31	I sport regularly.
32	I try to have a balanced diet.
33	I choose food that is produced in an environmental friendly way.
34	I buy fruits and vegetables directly from the farmer.
35	Whenever possible, I choose fruits and vegetables from my own allotments (plots).
36	I avoid buying food transported by aircrafts.
37	I buy locally produced foods.
38	I choose products that are not transported over long distances.
39	I try to eat as much pulses as possible in order to reduce meat consumption.
40	Pulses replace meat in my cooking.
41	I try to eat as much plant–protein source food products as possible, e.g., pulses.
42	I avoid eating meat.
43	I choose free-range eggs.
44	I avoid buying battery eggs.
45	Whenever possible I buy free range eggs.
46	Whenever possible, I buy fish from sustainable fishing.
47	I avoid food products containing lots of fat.
48	I choose low fat food products.
49	Whenever possible, I choose low fat food products.
50	I try not to throw away food.
